# Reducing surgical instrument usage: systematic review of approaches for tray optimization and its advantages on environmental impact, costs and efficiency

**DOI:** 10.1093/bjsopen/zraf030

**Published:** 2025-05-17

**Authors:** Myrthe M M Eussen, Esmee Logghe, Stijn Bluiminck, Daan J Comes, Merel L Kimman, Brigitte A B Essers, Lianne M Wellens, Schelto Kruijff, Philip R de Reuver, Nicole D Bouvy

**Affiliations:** Department of Surgery, Maastricht University Medical Center, Maastricht, The Netherlands; NUTRIM School of Nutrition and Translational Research in Metabolism, Maastricht University, Maastricht, The Netherlands; Department of Surgery, Maastricht University Medical Center, Maastricht, The Netherlands; Department of Surgery, Radboud University Medical Center, Nijmegen, The Netherlands; Department of Surgery, Radboud University Medical Center, Nijmegen, The Netherlands; Department of Clinical Epidemiology and Medical Technology Assessment (KEMTA), Maastricht University Medical Centre, Maastricht, The Netherlands; Department of Clinical Epidemiology and Medical Technology Assessment (KEMTA), Maastricht University Medical Centre, Maastricht, The Netherlands; NUTRIM School of Nutrition and Translational Research in Metabolism, Maastricht University, Maastricht, The Netherlands; Department of General Practice, Amsterdam University Medical Center, Amsterdam, The Netherlands; Department of Surgery, University Medical Center Groningen, Groningen, The Netherlands; Department of Surgery, Radboud University Medical Center, Nijmegen, The Netherlands; Department of Surgery, Maastricht University Medical Center, Maastricht, The Netherlands; NUTRIM School of Nutrition and Translational Research in Metabolism, Maastricht University, Maastricht, The Netherlands

## Abstract

**Background:**

Operating rooms generate substantial waste and budget expenditure due to extensive material usage. Reusable instruments are often packaged in trays, which accumulate instruments over time. This review quantifies the advantages of tray optimization (removing redundant instruments), including reduced environmental impact, costs, operating room and processing time.

**Methods:**

Following PRISMA guidelines, searches were conducted in PubMed, Embase, Web of Science and The Cochrane Library in August 2024 for studies on optimizing surgical trays in human surgeries. Studies were included if they reported on optimization approaches and outcomes related to environmental, economic or efficiency improvements. Exclusions included studies on disposable instruments, animal or veterinary research and patient-specific trays. Risk of bias was assessed using the ROBINS-I (Risk Of Bias In Non-randomised Studies - of Interventions) tool.

**Results:**

The search identified 4511 studies, with 45 meeting the inclusion criteria. Half of the studies showed a serious risk of bias, while the rest had a moderate risk. Three main optimization strategies were identified, with expert analysis being the most common (*n* = 29), followed by mathematical modelling. Environmental benefits were reported in all three included studies, although limited in number. Studies reported that 19 to 89% of instruments could be removed from trays, with 31 studies unanimously reporting cost reductions. Additionally, 17 studies demonstrated improved operational efficiency.

**Conclusion:**

Tray optimization strategies effectively reduce resource use, resulting in environmental and economic benefits. Although no standard method exists, effective strategies such as procedure observation and clinician feedback may eliminate over half of the instruments, offering a significant opportunity to minimize resource consumption in the operating room.

## Teaser

Optimizing surgical trays by removing redundant instruments can reduce environmental impact, costs and improve efficiency. This review outlines strategies for effective tray optimization, offering substantial resource savings.

## Introduction

Modern healthcare faces significant challenges, including rising costs, growing demand for services and space, and reducing the environmental impact^1^. These challenges put the viability of public health administration at risk. Reports from the World Health Organization (WHO) and the Organisation for Economic Co-operation and Development (OECD) show that healthcare spending accounted for 8.8% of the gross domestic product (GDP) in 2019, rising to 9.7% in 2021^2,3^. The sector is also responsible for 4.4% of net global emissions, with figures ranging from 6 to 8% in Western countries^4^. This sector consumes substantial resources, as demonstrated by the Dutch healthcare system, which uses 13% of the nation’s total resources^5^. This necessitates systemic reforms to enhance environmental sustainability, efficiency and cost-effectiveness in healthcare.

The operating room (OR) is a key area for reform, being one of the most resource-intensive hospital departments. It generates 20–30% of hospital waste and accounts for 30–60% of total costs^6–10^. Reusable instruments contribute considerably to the carbon footprint due to their cleaning and sterilization process, which accounts for 90% of the total environmental impact^11,12^. Surgical trays, containing reusable instruments packaged for specific procedures, represent one of the most frequently utilized materials in ORs. Over time, these trays evolve as instruments are added, even though may be seldom-used. Removing these seldom-used instruments could reduce the tray’s contents by 30–60%^13–18^. Therefore, tray optimization—by eliminating unnecessary or redundant instruments per tray—reduces ecological and economic impact, and improves procedural efficiency and standardization of the procedure.

Despite growing interest, optimizing surgical trays remains relatively underexplored in medical literature. In 2021, Dos Santos *et al*. outlined methods for analysing instrument usage, yet implementation in clinical settings is still challenging and far from standard^18^. This systematic review provides a comprehensive overview of strategies for optimizing surgical trays, offering practical guidance for surgeons on tray optimization and evaluating its potential benefits. It explores methods for assessing instrument usage, and evaluates appropriate thresholds for instrument removal. Additionally, this review quantifies the advantages of tray optimization, including reductions in waste, costs, OR time and processing time. Successful implementation of these strategies requires close collaboration between surgeons, OR management and sterilization departments. By synthesizing current evidence, this review aims to support the integration of tray optimization into routine hospital practices, highlighting the crucial role of the surgical team in alleviating burdens on healthcare systems.

## Methods

### Study protocol and registration

This systematic review was conducted in accordance with the 2020 PRISMA (Preferred Reporting Items for Systematic Reviews and Meta-Analyses) and SWiM (Synthesis Without Meta-analysis) guidelines^19,20^.

### Search strategy

In August 2024, a comprehensive search was performed across PubMed, Embase (via Ovid) and The Cochrane Library. The focus was on four key domains: reusable instrument trays; optimization strategies; implementation within surgical settings; and outcomes related to environmental and economic impact (including efficiency, such as operating room time). Search terms combined Medical Subject Headings (MeSH), Emtree and free-text phrases using AND-OR operators. No restrictions regarding the date of publication were applied. The detailed search strategy is available in the *Supplementary material*. Manual searches of references from relevant systematic reviews were performed to identify further studies.

### Selection process

Eligible studies were those that compared surgical trays before and after implementation of an optimization strategy. Trays with disposable products were excluded because these often included complete procedure trays containing not only surgical instruments but also other items like gowns and drapes. Studies describing the average usage rate of the instruments without creation of an optimized tray were excluded. Tray optimization was defined as the elimination of unnecessary or redundant instruments from each tray. Additionally, exclusions applied to studies on animal subjects, veterinary research, instruments not used in the operating room, patient-specific instrument trays, and secondary literature such as technical reports and conference proceedings. No restrictions on language were applied.

Duplicate records were removed, and two reviewers (M.E., E.L.) independently screened the titles and abstracts using the Rayyan tool^21^. Discrepancies were resolved through consensus meetings. Studies that met the inclusion criteria underwent full-text review. The EndNote Reference Management Tool™ was utilized to manage references throughout the review process.

### Data extraction

Data extraction was independently performed by two reviewers (M.E., E.L.) and included variables such as author(s), publication year, country, surgical procedures, tray types, methods for assessing instrument usage, thresholds for instrument elimination, primary objectives and main findings.

### Prioritization of studies and outcomes

Prioritization was guided by predefined criteria established before data extraction. Outcomes were prioritized based on their relevance to the review objectives, study quality and completeness of reported data. A structured table was used to extract and organize relevant outcomes from all included studies.

### Standardized metric and transformation methods used

Several metrics were selected to measure the impact of tray optimization, including instrument reduction (%), tray weight (kilograms or %), carbon footprint (CO₂ equivalent, kg CO₂eq), costs (euros or %) and time savings (minutes). These metrics, reflecting environmental and economic outcomes, were obtained from studies or manually calculated by two reviewers (M.E., E.L.) if percentages were not reported. Discrepancies in reported units, such as local currencies or weights, were standardized to euros ($1:€0.95, conversion date 26 January 2025) and kilograms for consistency.

### Risk-of-bias assessment

The risk of bias was independently assessed by two reviewers (M.E., E.L.) using the Cochrane ROBINS-I (Risk Of Bias In Non-randomised Studies - of Interventions) tool^22^. An arbitrator (L.W.) was available to resolve any disagreements between the reviewers during the selection and assessment phases.

### Analysis

Data from the studies were synthesized and presented in tabular form, accompanied by a narrative description of the findings. Due to significant variability among the included studies, performing a meta-analysis was not viable.

## Results

### Study selection

**Fig. 1:**
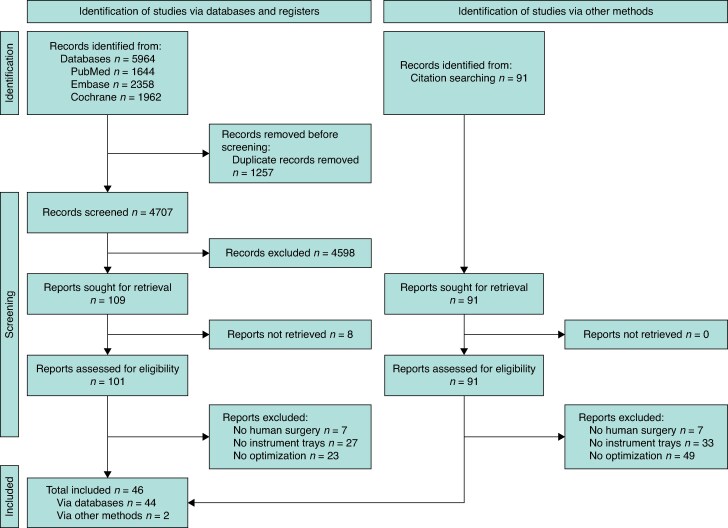
PRISMA flow chart outlining the strategy for study selection

After removing duplicates, the search identified 4707 studies. An initial screening of titles and abstracts narrowed this to 109 potential studies. Full-text assessment was not possible for eight studies due to unavailability, leaving 101 studies for further evaluation. Of these, 44 met the inclusion criteria based on the full-text review. Additionally, 91 references were checked, leading to the identification of two more eligible studies. In total, 46 studies were included in this review (see *Fig. 1*).

### Study characteristics

The 46 included studies encompassed a wide range of surgical specialties (see *Table 1*). The majority of the studies focused on otorhinolaryngology (*n* = 10)^15,16,23–30^ and orthopaedic surgery (*n* = 8)^31–39^. Cardiothoracic and vascular trays were optimized in six studies^40–45^. Gynaecological^12,46–48^, paediatric^13,49–51^ and plastic surgical trays^52–55^ were each examined in four studies, while three studies were devoted to breast surgery^56–58^ and neurosurgery^59,60^. Two studies investigated ophthalmology^61,62^. Additionally, one study focused on urology^14^. Two studies by Toor *et al*. examined a combination of procedures from different specialties, each using a variety of trays^48,63^. The first study investigated a combination of otorhinolaryngology, general, gynaecologic and orthopaedic surgical trays^63^, while the second study focused on trays used in general surgery, gynaecology and gynaecological oncology^48^.

**Table 1:** Characteristics of included studies (*n* = 46)

Author	Year	Country	Surgical procedure(s)	Name of instrument tray(s)	Aim of the study
**Breast surgery (*n* = 3)**					
Holland *et al*.^56^	2022	USA	Excisional biopsy, lumpectomy, lymph node sampling and mastectomy	General surgery	To evaluate cost savings
Malone *et al*.^57^	2019	USA	Lumpectomy	Head and neck tray (used for lumpectomy)	To evaluate cost savings and efficiency improvement
Schwartz *et al*.^58^	2021	USA	Excisional biopsy and mastectomy	NA	To evaluate cost savings
**Cardiothoracic and vascular surgery (*n* = 6)**					
Barua *et al*.^42^	2017	USA	CABG	CABG tray	To evaluate efficiency improvement
Friend *et al*.^40^	2018	USA	VATS	Basic thoracic, chest and retractor tray	To evaluate efficiency improvement
Knowles *et al*.^43^	2021	USA	NA	Vascular tray and aortic tray	To evaluate efficiency improvement
Sanchez *et al*.^41^	2023	USA	NA	Robotic and conventional laparoscopic/thoracic tray	To evaluate cost savings
Taylor *et al*.^44^	2020	USA	Transcarotid artery revascularization	General vascular tray	To evaluate use of instruments in trays
Warner *et al*.^45^	2015	USA	Endovascular aneurysm repair	Generic small vessel and minor vascular tray	To evaluate cost savings
**Gynaecologic surgery (*n* = 3)**					
Bachmann *et al*.^46^	1998	USA	NA	Laparoscopic tubal ligation, laparoscopic diagnostic and laparoscopic operative procedure tray	To evaluate cost savings
Harvey *et al*.^47^	2017	USA	NA	Vaginal nr. 1, vaginal nr. 2, gynaecology laparoscopy, gynaecology minimally invasive and gynaecology pelvic support specialty tray	To evaluate cost savings
Schmidt *et al*.^12^	2023	NL	Abdominal radical hysterectomy	Wertheim-, gynaecologic abdominal and basis large tray	To evaluate environmental impact
**Neurosurgery (*n* = 3)**					
Belhouari *et al*.^59^	2023	Canada	Laminectomy	Laminectomy and basic neurosurgery	To evaluate cost savings and efficiency improvement
Farrokhi *et al*.^60^	2015	USA	DBS, craniotomy, minimal invasive and open spine surgery	NA	To evaluate cost savings and efficiency
Lunardini *et al*.^37^	2014	USA	Anterior cervical and lumbar discectomy, foraminotomy, lumbar interbody fusion, lumbar laminectomy and fusion	Generic spine instrument tray	To evaluate cost savings
**Ophthalmology (*n* = 2)**					
Grodsky *et al*.^61^	2020	USA	Vitrectomy	NA	To evaluate cost savings
Schneider *et al*.^62^	2020	Brazil	NA	Basic ophthalmology, cataract surgery, chalazion, dacryo, enucleation, glaucoma, lacrimal dilators, large ophthalmic plastic, peribulbar block, phacoemulsification, pterygium, retina micro, retinopexy, small ophthalmic plastic, strabismus and vitrectomy tray	To evaluate use of instruments in trays
**Orthopaedics (*n* = 8)**					
Adamczyk *et al*.^31^	2022	Canada	Total hip arthroplasty	Standard total hip arthroplasty tray	To evaluate cost savings, efficiency improvement and reduction in waste
Capra *et al*.^32^	2019	USA	Total knee arthroplasty, and total hip arthroplasty	Total knee arthroplasty, and total hip arthroplasty tray	To evaluate cost savings and efficiency improvement
Cichos *et al*.^33^	2019	UK	Total joint arthroplasty	Shoulder retractors, shoulder, shoulder scope, scope, total knee, foot and ankle specials, foot, hand and elbow, hand and elbow bone, soft tissue hand, simple hand, shoulder specials and anterior cruciate ligament	To evaluate efficiency improvement
Helmkamp *et al*.^34^	2022	USA	Carpometacarpal arthroplasty	Hand and foot	To evaluate cost savings
Hermena *et al*.^35^	2021	UK	Hip, knee and shoulder procedures, dynamic hip screw fixation, internal fixation, proximal humerus fracture reduction, shoulder arthroplasty, shoulder rotator cuff repair and shoulder stabilization	NA	To evaluate cost savings
Lonner *et al*.^36^	2021	USA	Total knee arthroplasty, and total hip arthroplasty	NA	To evaluate cost savings
Parker *et al*.^38^	2024	USA	Foreign body removal, gastrocnemius recession, hammertoe correction, mass excision and toe amputation	Hand and foot tray	To evaluate environmental impact
Toor *et al*.^39^	2021	Canada	Total knee arthroplasty, total hip arthroplasty trauma and orthopaedic oncology	Major orthopaedic tray	To evaluate cost savings
**Otorhinolaryngology (*n* = 10)**					
Chin *et al*.^17^	2014	Canada	Endoscopic sinus surgery, septoplasty, septorhinoplasty, skin cancer excision and tonsillectomy	OHNS trays	To evaluate use of instruments in trays
Crosby *et al*.^16^	2020	Canada	Sinus surgery, septoplasty, septorhinoplasty and tonsillectomy	OHNS trays	To evaluate cost savings and efficiency improvement
Dyas *et al*.^23^	2018	USA	Parathyroidectomy and thyroidectomy	Head and neck trays	To evaluate cost savings and efficiency improvement
Fu *et al*.^24^	2021	Canda	Adenotonsillectomy, endoscopic sinus surgery, myringotomy, septoplasty and thyroidectomy	NA	To evaluate cost savings and efficiency improvement
Gidumal *et al*.^25^	2021	USA	Rhinoplasty	Rhinoplasty, rhinology and oculoplasty tray	To evaluate cost savings
John-Baptiste *et al*.^26^	2016	Canada	Endoscopic sinus surgery, septoplasty, septorhinoplasty, skin cancer excision and tonsillectomy	NA	To evaluate cost savings
Van Osch *et al*.^30^	2024	Canada	Posttonsillectomy haemorrhage and peritonsillar abscess	Tonsillar haemorrhage tray	To evaluate cost savings and efficiency improvement and environmental impact
Wannemuehler *et al*.^27^	2015	USA	Adenotonsillectomy	Adenotonsillectomy tray	To evaluate cost savings and efficiency improvement
Yalamanchi *et al*.^28^	2022	USA	Multiple head and neck procedures	Major otolaryngology, oto plastics, direct laryngoscopy and microdirect laryngoscopy tray	To evaluate cost savings
Yoon *et al*.^29^	2019	USA	NA	OHNS tray	To evaluate use of instruments in trays
**Paediatric surgery (*n* = 4)**					
Farrelly *et al*.^14^	2017	USA	NA	General paediatric surgery trays	To evaluate cost savings and efficiency improvement
Herlihy *et al*.^49^	2023	Ireland	Inguinal hernia repair and inguinoscrotal orchidopexy	Inguinoscrotal tray	To evaluate cost savings and efficiency improvement
Koyle *et al*.^50^	2018	Canada	Inguinal hernia repair	Hernia tray	To evaluate efficiency improvement
Shaw *et al*.^51^	2022	USA	Hydrocelectomy, inguinal hernia repair and orchiopexy	GU minor, GU specials, GU major, GU orchipexy, GU meatotomy, GU circumcision	To evaluate cost savings and efficiency improvement
**Plastic surgery (*n* = 4)**					
Dorante *et al*.^52^	2023	USA	Reduction mammoplasty	NA	To evaluate cost savings
Kirn *et al*.^53^	2018	USA	Carpal tunnel release, extensor compartment release, ganglion cyst excision and trigger finger or thumb release	Hand tray	To evaluate cost savings
Kodumuri *et al*.^54^	2023	UK	Carpal tunnel release	Carpal tunnel release tray	To evaluate cost savings and environmental impact
Wood *et al*.^55^	2021	USA	NA	General plastics, and breast reconstruction tray	To evaluate cost savings
**Urology (*n* = 1)**					
Nast *et al*.^15^	2019	USA	Hernia repair and orchiopexy	Minor urology tray	To evaluate cost savings
**Other (*n* = 2)**					
Toor *et al*.^63^	2022	Canada	Multiple procedures in ENT, general, gynaecologic and orthopaedic surgery	ENT: ENT and neck accessory tray General: breast extras, breast, lap chole, laparoscopic, major general surgery, minor general surgery, and perineal tray Gynaecologic: abdominal hysterectomy, gynae laparoscopic, gynae onc laparoscopic, and vaginal hysterectomy Orthopaedic: hip accessory, major ortho, minor ortho and tumour tray	To evaluate cost savings and efficiency improvement
Toor *et al*.^48^	2022	Canada	NA	General surgery, gynaecology and gynaecological oncology trays	To evaluate cost savings

CABG, coronary artery bypass graft; DBS, deep brain stimulation; ENT, otorhinolaryngology; GU, genitourinary; NA, not available; NL, The Netherlands; OHNS, otorhinolaryngology; VATS, video-assisted thoracoscopic surgery.

Among the included studies, all reported the number of instruments in the trays before and after optimization as well as the optimization methods employed. The majority of these studies (*n* = 42) described the impact of the reduction further in terms of environmental outcomes or costs.

### Strategies used for optimizing surgical trays

#### Approach and technique

Three approaches for optimizing surgical trays were identified in the included studies^1^: expert analysis (*n* = 29), mathematical programming (*n* = 5) and lean practices (*n* = 11) (see *Table S1*). In one study, the approach used to optimize the trays was not described^38^.

Within the expert analysis category, various techniques were described. In the majority of studies (*n* = 13), this involved staff reviewing the instruments in the tray and removing those that seemed unnecessary based on their opinion^13,29,30,32,34,40,41,44,46,53,56–58,61^. Surgeons led this review, often in collaboration with surgical technicians, scrub nurses, or personnel from material management and the central or sterile processing departments. In one study, this review process was followed by a meeting to discuss the data and reach a consensus^47^. In seven studies, the actual usage of the instruments was observed during surgical procedures to determine which instruments to include or exclude^15,16,26,34,42,49,52^. In five studies, these observations were combined with reviews by surgeons^24,25,28,31,63^, and in two studies, consensus meetings were held after the observations^23,47^.

Mathematical programming was applied in five studies, employing a linear model to determine the most optimal tray configuration^12,39,48,59,62^. In one of these studies, surgeries were observed to determine the usage rate of each instrument, and this data was integrated into a linear programming model to reduce the number of instruments in the tray^12^. In two studies, observations and staff reviews were used as data inputs for the linear model^48,62^. Toor *et al*. also included a consensus group to validate the model results before eliminating instruments from clinical practice^39^. A study by Belhouari *et al*. combined three methods: mathematical modelling based on usage rate observations, staff reviews and a mathematical model incorporating procedure observations in association with cost-inflection point analysis^59^.

Eleven studies specifically mentioned the application of the lean methodology, designed to reduce non-value-adding activities by following five key steps: defining value; mapping the value stream; creating flow; implementing a pull system and pursuing perfection^14,27,33,36,37,43,45,50,54,55,60^. This involved observing actual instrument use during procedures, analysing the data, presenting the outcomes to faculty staff, reviewing of the set by the staff and/or meetings to reach a consensus on which instruments to remove. In one study, the specific actions taken in the lean practices were not described^54^.

#### Number of observations or reviews

Of the 46 included studies, 27 reported the number of observed surgical procedures or reviews by clinicians, with sample sizes ranging from 6 to 1500 and a median of 82 (see *Table S1*).

#### Cut-off value

The majority of the studies do not describe the cut-off value or the criteria used to determine whether an instrument would remain on the tray (*n* = 26). In the studies where the actual usage of instruments was observed during surgical procedures, whether or not as part of lean practices, 13 studies defined the cut-off value based on the instrument usage rate (IUR) for removing instruments from the trays. The cut-off values ranged from 12.5 to 50% IUR. In most studies (*n* = 7), instruments were removed from the tray if they were used in less than 20% of the observed cases^12,14–16,25,33,52^.

In the studies involving staff reviews, only five studies specified the criteria for instrument removal. In four of these five studies, unanimous agreement among clinicians was required for removal of an instrument^13,30,36,39^. In the study by Belhouari *et al*., agreement from at least half of the staff faculty members was necessary^59^.

### Outcomes of optimized surgical trays

#### Instrument reduction, utilization rate and tray weight

**Fig. 2:**
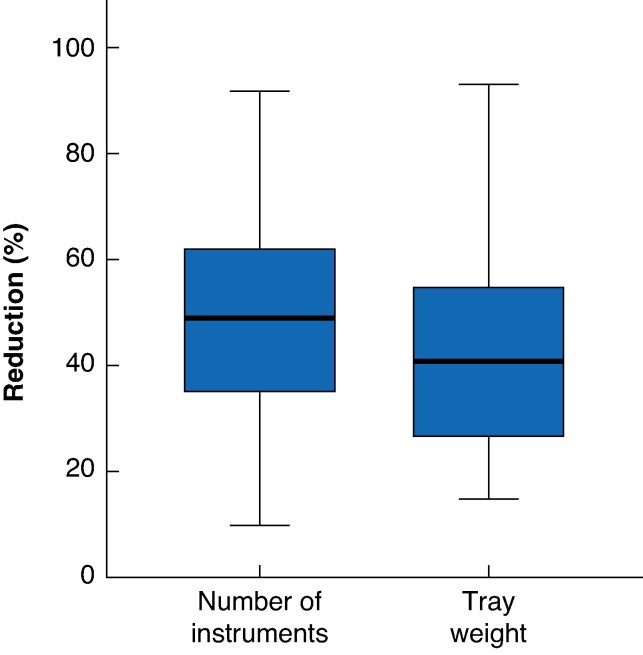
Percentage reduction in the number of instruments and tray weight per tray after optimization

The included studies identified a potential reduction in the number of instruments of 19–89% (see *Table S2*, *Fig. 2*). The majority of studies indicated that over 50% of the instruments could be removed from the trays (*n* = 20). Eleven studies reported a possible reduction of 40–50%. Three studies found that less than 20% of the instruments could be removed.

In nearly one-third of the studies (*n* = 14), the mean IUR before optimization was reported, ranged from 16 to 58% (see *Table S2*). Of these, four studies calculated the usage rate after optimization, which increased to between 47% and 80% (see *Table S2*).

Twelve studies examined the weight per tray before and after optimization, resulting in an average weight decrease of 43%, with reductions ranging from 15 to 93% (see *Table S2*, *Fig. 2*).

#### Environmental impact

The environmental impact was described in four studies (see *Table 1*)^12,31,54^. Two studies conducted a life cycle assessment (LCA), analysing every aspect of the product cycle, including cleaning and sterilization. Schmidt *et al*. conducted an LCA and estimated that, before optimization, the carbon footprint of a surgical tray used in abdominal radical hysterectomy was 6.41 kg CO_2_eq (95% c.i. 5.87–7.30) per procedure. Three key factors contributed to this emission: wrapping paper used during sterilization (3.82 kg CO_2_eq), energy consumed by the washer-disinfector (1.92 kg CO_2_eq) and energy used during steam sterilization (0.63 kg CO_2_eq). By reducing the number of instruments in the tray by 29% (from 154 to 109 instruments) and decreasing the tray size, the carbon footprint was reduced by 2.22–3.7 kg CO_2_eq per procedure^12^. Kodumuri *et al*. reduced the number of instruments for a carpal tunnel release from 25 to 7 (−72%), corresponding to a decrease of 9.4 kg CO_2_eq per procedure (−66%) (preoptimization: 14.1 kg CO_2_eq; postoptimization: 4.7 kg CO_2_eq). The emissions from this tray were also primarily attributed to the cleaning and sterilization cycle^54^.

Van Osch *et al*. estimated that reducing the instruments in the tonsil haemorrhage tray by 44% could lower the CO_2_eq from 6.11 kg to 2.85 kg per year, based on previous literature^30^. Adamczyk *et al*. identified a reduction of 7.16 kWh of energy consumption and 1.61 kg of waste per case, primarily generated by the decrease in sterile wrapping and drapes, by reducing the number of instruments by 49% in the standard total hip arthroplasty tray^31^.

#### Costs and efficiency

**Fig. 3:**
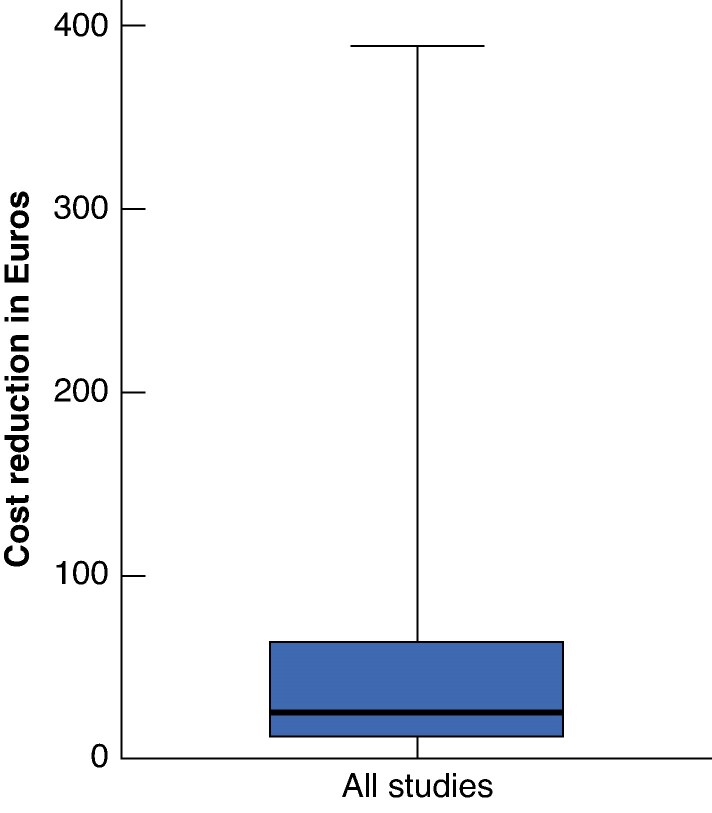
Cost reduction in euros per tray after optimization

Among the studies included, 32 examined the economic impact of tray optimization. All studies reported the cost reductions, ranging from €0.19 to €387.78 per procedure (see *Table 1*, *Fig. 3*). Only nine studies described the actual costs before and after optimization, and presented relative cost reductions. Relative cost reductions ranged from 32 to 78%^27,34,35,41,46,54,57^. Notably, the factors included in these cost analyses vary significantly between the studies (see *Table 1*).

Nineteen studies (39%) described changes in efficiency in terms of tray set-up time, surgical time, total OR time, time necessary to count the instruments, turnover time, cleaning time and assembly time (see *Table 1*)^13,15,23,24,27,30–33,36,40,42,43,49–51,54,60^. Twelve studies specifically addressed the change in set-up time, with half of these studies demonstrating a significant decrease in the mean set-up time^15,27,31,32,36,41,43,50,51,60^. Optimizing surgical trays resulted in a reduction of total OR time by 7–39 min^54,60^. On the other hand, Adamczyk *et al*. found no significant reduction in surgical and total OR time before and after the optimization of an orthopaedic tray^31^. Barua *et al*. and Herlihy *et al*. reported reductions of 76 and 42 s in time to count instruments respectively, following an instrument reduction of 34 and 35%^42,49^. Cichos *et al*. documented a reduction in mean turnover time from 39.3 min to 38.4 min after eliminating 55% of the instruments in multiple trays used for total joint arthroplasty^33^. Four studies investigated cleaning time, with all revealing no significant decreases^30,32,33,36^. A significant reduction in assembly time was found in half of the studies reporting on OR time (*n* = 2), with one study showing a 58–66% decrease and another reporting a reduction of 3.7 min^15,27^.

### Risk of bias

**Fig. 4:**
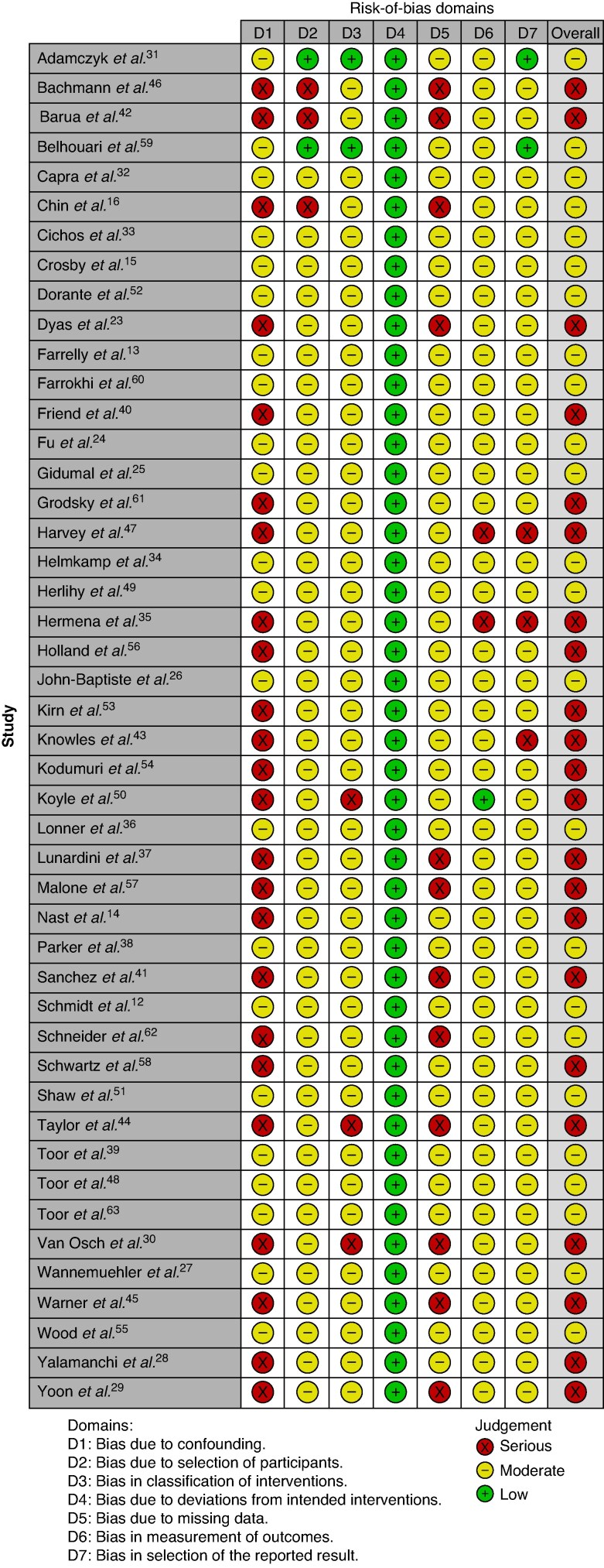
Risk-of-bias assessment of the included studies by ROBINS-I (Risk Of Bias In Non-randomised Studies - of Interventions) tool^22^

Included studies exhibited serious risk in 22 cases and moderate risk in 24 cases (see *Fig. 4*). Studies generally lacked sufficient reporting on critical elements, such as methods for controlling bias, and selection of participants.

## Discussion

This review highlights the potential of surgical tray optimization to reduce resource use, resulting in both environmental and economic benefits. While numerous strategies have been outlined in the literature, high-quality studies to identify the most effective methods are still lacking. Procedure observation and surgeon feedback are frequently cited as effective techniques for eliminating unused instruments, with some studies reporting reductions in tray contents by more than half. Although surgeons play a key role in these efforts, the involvement of the broader surgical team is crucial to overcoming the barriers to implementing such changes.

In healthcare, particularly in ORs, substantial material consumption places a considerable environmental and financial strain on the sector^2–5^. Streamlining OR processes presents an opportunity to mitigate this impact and achieve cost savings. Many surgical instruments are available in both reusable and single-use variants, and addressing the challenges requires systemic reform. Surgical trays often contain numerous instruments, many of which remain unused during procedures^13–18^. Sterilizing these unused instruments imposes an unnecessary resource burden. Studies included in this review suggest that reducing the number of instruments in trays is a promising strategy to lower both environmental impact and costs. Given the contribution of ORs to overall hospital resource consumption, optimizing trays could serve as an important initial step in broader resource management efforts across healthcare systems^13–18^.

This review of 46 studies found considerable variation in tray optimization methods, including expert analysis, mathematical modelling and lean practices, each offering advantages and limitations. Hospitals should assess their available resources to determine the most feasible and appropriate approach for their specific circumstances. Most studies reported removing instruments used in less than 20% of procedures, which can lead to reductions in carbon footprint and costs. Despite the diversity of methods, observing procedures or having clinicians review sets are frequently cited as relatively simple, quick, inexpensive and easily applicable methods.

A significant variation in IUR was also observed, with many studies noting that more than half of the instruments in trays could be removed due to infrequent use. Reducing the number of instruments not only simplifies the trays but also decreases their weight. Even before considering the environmental and economic benefits— which should be investigated together in future research considering the entire life cycle of a product—this practice should be routinely integrated into healthcare procedures.

Environmental considerations are vital in clinical practice, and while few studies evaluate them, all show clear benefits from reducing the number of instruments^64^. The environmental impact of reusable instruments is largely driven by the sterilization process, and optimizing trays can reduce the carbon footprint by 40–66%^12,65^. Smaller tray sizes, determined by the number and size of instruments, allow more trays to be cleaned and sterilized at once, further minimizing environmental impact. This reduction aligns with the 10R circular economy model—refuse, reduce, rethink, reuse and more—which prioritizes actions that lower the environmental footprint of products^66^. To fully realize these benefits, reducing instrument numbers must be paired with tray size adjustments. Effective implementation of this strategy requires close collaboration between surgeons and the sterilization department to ensure trays are optimized for both clinical efficiency and environmental sustainability.

Although environmental considerations are unconditional in clinical practice, the cost of our healthcare system remains a critical concern, making cost-reduction measures highly valued in clinical practice^64^. Regarding economic outcomes, cost savings from tray optimization varied widely, ranging from 32 to 78%, due to differences in the factors considered in these calculations. Tray optimization reduces costs not only by minimizing the number of instruments used but also by improving efficiency in set-up and postprocedure processes^15,27,31–33,36,41–43,49–51,54,60^. These efficiency improvements, including reductions in mean set-up time, counting time and turnover time, suggest potential for personnel cost savings. Despite variations in methodologies and study intervals (1998 to 2024), all reviewed studies consistently demonstrated cost savings, primarily through reductions in material usage and process-related expenses.

In addition to the benefits of tray optimization in terms of cost, environmental sustainability and efficiency, it is important to consider the potential impact on procedures and patient outcomes if a removed instrument is missing. This impact is probably minimal, especially as good alternatives are likely available. Most studies have not addressed this aspect. Instruments with an IUR of less than 20% may not be missed by the operator, thus having little to no impact on patient safety. Therefore, it might be concluded that tray optimization positively impacts environmental and economic outcomes without compromising patient safety.

Surprisingly, all surgical specialties were well represented across multiple studies, except for general and abdominal surgery, which were investigated in only two studies and in combination with other surgical specialties. However, set optimization within this specialty also deserves attention, especially given the large amounts of reusable materials available in various variants. For example, a wide variety of retractors and forceps are typically on hand during these procedures. The results and benefits of set optimization identified in other surgical fields can likely be easily extrapolated to general and abdominal surgery due to the extensive range of studies described.

This study acknowledges several limitations, primarily related to the low to moderate quality of the included studies. Given the limited availability of high-quality literature and insufficient reporting on critical elements such as methods for controlling bias and participant selection, the authors still chose to include these studies in the review despite these shortcomings. Heterogeneity in methods, study designs and outcomes across the studies prevented the possibility of conducting a meta-analysis. The absence of standardized assessment tools, coupled with the inclusion of various surgical subspecialties and differing tray types, may have introduced variability in the reported results. Most studies presented economic outcomes as absolute savings following tray optimization. However, expressing these cost reductions as percentages relative to the original costs would offer a more accurate measure of effectiveness.

When permanently removing instruments from sets, it is important to consider what should be done with these items. Should they be kept available for exceptional cases, or offered in disposable or reusable forms, given that sterilization heavily influences the environmental footprint of reusable instruments? The impact of these decisions should be further investigated. Additionally, in situations where only a portion of the instruments is removed, the risk arises that a new, fully stocked tray might need to be opened just to access one missing instrument. To mitigate this, hospitals could consider packaging certain instruments individually in sterile wrapping, allowing them to be accessed without opening an entirely new tray. Future studies should explore these aspects more extensively to reflect actual clinical use, not only the tray itself but also any material that needs to be separately packaged and/or opened to compensate for the instruments removed from the tray.

Despite the absence of a fixed method for optimizing trays, it can be concluded that tray optimization offers significant advantages in terms of environmental and economic outcomes. On average, more than half of the instruments can be removed from trays in the majority of cases. Various methods for identifying these instruments exist, with observing actual use during procedures or reviewing sets by clinicians being effective starting points. The benefits of tray optimization outweigh the potential drawbacks, providing a clear path forward for improving resource use in the operating room.

## Supplementary Material

zraf030_Supplementary_Data

## Data Availability

Data sets generated during and/or analysed during the present study are available upon reasonable request.
