# Supplementary material for: Reducing surgical instrument usage: systematic review of approaches for tray optimization and its advantages on environmental impact, costs and efficiency
Source: BJS Open. 2025 May 17;9(3):zraf030. doi: 10.1093/bjsopen/zraf030 (PMC12084675; doi:10.1093/bjsopen/zraf030)
Supplement: zraf030_Supplementary_Data [file zraf030_supplementary_data.docx]

**Reducing surgical instrument usage: a systematic review of approaches for tray optimization and its advantages on environmental impact, costs and efficiency**

Myrthe M.M. Eussen MD^1,2^, Esmee Logghe BSc^1^, Stijn Bluiminck MD ^3^, Daan J. Comes MD ^3^, Merel L. Kimman PhD^4^, Brigitte A.B. Essers PhD^4^, Lianne M. Wellens MD PhD^1,5^, Schelto Kruijff MD PhD^6^, Philip R. de Reuver MD PhD^3^, Nicole D. Bouvy MD PhD^1,2^

^1^ Department of Surgery, Maastricht University Medical Center, Maastricht, The Netherlands

^2^ NUTRIM School of Nutrition and Translational Research in Metabolism, Maastricht University, Maastricht, The Netherlands

^3^ Department of Surgery, Radboud University Medical Center, Nijmegen, The Netherlands

^4^ Department of Clinical Epidemiology and Medical Technology Assessment (KEMTA), Maastricht University Medical Centre, Maastricht, The Netherlands

^5^ Department of General Practice, Amsterdam University Medical Center, Amsterdam, The Netherlands

^6^ Department of Surgery, University Medical Center Groningen, Groningen, The Netherlands

**Corresponding author.**

Prof. dr. N. D. Bouvy

Department of Surgery, Maastricht University
Medical Centre

PO Box 5800, 6202 AZ Maastricht, the Netherlands

E-mail: n.bouvy@mumc.nl

https://orcid.org/0000-0003-4070-8559

**Supplementary Materials - Index**

| **Supplementary Methods** |  |
| --- | --- |
| Search strategy | *Page 3* |
| **Supplementary Results**  Table S1: Strategies for optimizing surgical trays  Table S2: Effects of tray optimization on number of instruments, instrument usage and weight | *Page 10*  *Page 15* |
|  |  |

**Supplementary Methods**

**Supplementary: search strategy**

| ***Database*** | ***Search syntax*** |
| --- | --- |
| Pubmed | ((((("Surgical Instruments"[Mesh]) OR (instrument*[Title/Abstract])) OR (tray*[Title/Abstract])) AND (((((optimisation[Title/Abstract]) OR (optimization[Title/Abstract])) OR (optimising[Title/Abstract])) OR (reduction[Title/Abstract])) OR (reducing[Title/Abstract]))) AND ((((operating room[MeSH Terms]) OR (surgical procedures, operative[MeSH Terms])) OR ("operating room*"[Title/Abstract])) OR (surg*[Title/Abstract]))) AND (((((((((("Costs and Cost Analysis"[Mesh]) OR ("Efficiency"[Mesh])) OR (cost*[Title/Abstract])) OR (economic*[Title/Abstract])) OR (saving*[Title/Abstract])) OR (efficacy[Title/Abstract])) OR ("life cycle assessment"[Title/Abstract])) OR (sustainab*[Title/Abstract])) OR (footprint*[Title/Abstract])) OR (environment*[Title/Abstract])) |
| Embase (via Ovid) | surgical equipment/ OR "instrument*".ab,kw,ti. OR "tray*".ab,kw,ti. AND optimisation.ab,kw,ti. OR optimization.ab,kw,ti. OR optimising.ab,kw,ti. OR optimizing.ab,kw,ti. OR reduction.ab,kw,ti. OR reducing.ab,kw,ti. AND operating room/ OR surgery/ OR "operating room*".ab,kw,ti. OR "surg*".ab,kw,ti. AND economic evaluation/ OR productivity/ OR "cost*".ab,kw,ti. OR "economic*".ab,kw,ti. OR "saving*".ab,kw,ti. OR efficacy.ab,kw,ti. OR "life cycle assessment".ab,kw,ti. OR "sustainab*".ab,kw,ti. OR "footprint*".ab,kw,ti. OR "environment*".ab,kw,ti. |
| The Cochrane Library | [Surgical Instruments] explode all trees OR (instrument*):ti,ab,kw OR (tray*):ti,ab,kw AND optimisation.ab,kw,ti. OR optimization.ab,kw,ti. OR optimising.ab,kw,ti. OR optimizing.ab,kw,ti. OR reduction.ab,kw,ti. OR reducing.ab,kw,ti. AND [Operating Rooms] explode all trees OR [Specialties, Surgical] explode all trees OR (operating room*):ti,ab,kw OR (surg*):ti,ab,kw AND [Costs and Cost Analysis] explode all trees OR [Efficiency] explode all trees OR (cost*):ti,ab,kw OR (economic*):ti,ab,kw OR (saving*):ti,ab,kw OR (efficacy):ti,ab,kw OR (life cycle assessment):ti,ab,kw OR (sustainab*):ti,ab,kw OR (footprint*):ti,ab,kw OR (environment*):ti,ab,kw |

**Supplementary Results**

**Supplementary Table S1: Strategies for optimizing surgical trays**

| ***Author*** | ***Year*** | ***Country*** | ***Approach*** | ***Technique*** | ***Number of observationsor reviews*** | ***Cut-off value for exclusion of instruments*** | ***Number of instruments (pre-op vs post-op)*** |
| --- | --- | --- | --- | --- | --- | --- | --- |
| *Breast surgery (n=3)* | | | | | | |  |
| Holland et al.(56) | 2022 | USA | EA | Reviewing by staff | 656 | NA | -21% |
| Malone et al.(57) | 2019 | USA | EA | Reviewing by staff | NA | NA | -48% |
| Schwartz et al.(58) | 2021 | USA | EA | Reviewing by staff | Pre-op: 239  Post-op: 210 | NA | -49% |
| *Cardiothoracic and vascular surgery (n=6)* | | | | | | | |
| Barua et al.(42) | 2017 | USA | EA | Observations of use during procedures | 16 | NA | -34% |
| Friend et al.(40) | 2018 | USA | EA | Reviewing by staff | NA | NA | -41% |
| Knowles et al.(43) | 2021 | USA | EA, LP | Observations of use during procedures; analyzing and presenting data; consensus groups | 201 | NA | -55% |
| Sanchez et al.(41) | 2023 | USA | EA | Reviewing by staff | NA | <50% IUR | -62% |
| Taylor et al.(44) | 2020 | USA | EA | Reviewing by staff | NA | NA | -92% |
| Warner et al.(45) | 2015 | USA | LP | NA | 127 | NA | -32% |
| *Gynecologic surgery (n=3)* | | | | | | | |
| Bachmann et al.(46) | 1998 | USA | EA | Reviewing by staff | NA | NA | -44% |
| Harvey et al.(47) | 2017 | USA | EA | Reviewing by staff; consensus groups | NA | NA | -32% |
| Schmidt et al.(13) | 2023 | NL | EA, MP | Observations of use during procedures; integer linear programming | 6 | <20% IUR | -29% |
| *Neurosurgery (n=3)* | | | | | | | |
| Belhouari et al.(59) | 2023 | Canada | EA, MP | Mathematical model based on observations of use during procedures; reviewing by staff; combination of mathematical model, observations and cost inflection point analysis | 83 | >50% of the staff agreed on removing; instrument for which cost-based point of infection exceeded the weighted average of necessity | -17% |
| Farrokhi et al.(60) | 2015 | USA | EA, LP | Observations of use during procedures; analyzing data and presenting; reviewing by staff | Pre-op: 20  Post-op: 30 | NA | -75% |
| Lunardini et al.(37) | 2014 | USA | EA, LP | Observations of use during procedures; analyzing and presenting data; reviewing by staff | 38 | NA | -41% |
| *Ophthalmology (n=2)* | | | | | | | |
| Grodsky et al. (61) | 2020 | USA | EA | Reviewing by staff | 189 | NA | -89% |
| Schneider et al.(62) | 2020 | Brasil | EA, MP | Observations of use during procedures; reviewing by staff; linear programming | 20 | NA | -21% |
| *Orthopedics (n=8)* | | | | | | | |
| Adamczyk et al.(31) | 2022 | Canada | EA | Observations of use during procedures; reviewing by staff | 80 | NA | -49% |
| Capra et al.(32) | 2019 | USA | EA | Reviewing by staff | Pre-op: 38 Post-op: 58 | NA | -57% |
| Cichos et al.(33) | 2019 | UK | EA, LP | Observations of use during procedures; reviewing by staff; consensus groups | 11 | <20% IUR | -55% |
| Helmkamp et al.(34) | 2022 | USA | EA | Observations of use during procedures | 8 | <12.5% IUR | -50% |
| Hermena et al.(35) | 2021 | UK | EA | Reviewing by staff | NA | NA | -62% |
| Lonner et al.(36) | 2021 | USA | EA, LP | Observations of use during procedures; analyzing and presenting data; reviewing by staff | 35 | Instruments with 0% usage rate were automatically removed; for the remaining instruments all clinicians had to agree on removal | -37% |
| Parker et al.(38) | 2024 | USA | NA | NA | 40 | NA | -64% |
| Toor et al. (39) | 2021 | Canada | EA, MP | Observations of use during procedures; reviewing by staff; linear programming; consensus groups | 80 | All clinicians had to agree on removal | -42% |
| *Otorhinolaryngology (n=10)* | | | | | | | |
| Chin et al.(17) | 2014 | Canada | EA | Observations of use during procedures | 137 | <20% IUR | -58% |
| Crosby et al. (16) | 2020 | Canada | EA | Observations of use during procedures | Pre-op: 36 Post-op: 50 | <20% IUR | -52% |
| Dyas et al. (23) | 2018 | USA | EA | Observations of use during procedures; consensus groups | NA | NA | -63% |
| Fu et al.(24) | 2021 | Canda | EA | Observations of use during procedures; reviewing by staff | 238 | <25% IUR | -29% |
| Gidumal et al.(25) | 2021 | USA | EA | Observations of use during procedures; reviewing by staff | 10 | <20% IUR | -64% |
| John-Baptiste et al.(26) | 2016 | Canada | EA | Observations of use during procedures | NA | NA | -56% |
| Van Osch et al.(30) | 2024 | Canada | EA | Survey to staff on instrument usage | 55 | Instruments marked as “never used” by most respondents were eliminated, and duplicates were removed. | -44% |
| Wannemuehler et al.(27) | 2015 | USA | EA, LP | Observations of use during procedures; analyzing and presenting data; reviewing by staff | Pre-op: 51 Post-op: 61 | NA | -54% |
| Yalamanchi et al.(28) | 2022 | USA | EA | Observations of use during procedures; reviewing by staff | 1500 | <40% IUR | -28% |
| Yoon et al.(29) | 2019 | USA | EA | Reviewing by staff | NA | NA | -10% |
| *Pediatric surgery (n=4)* | | | | | | | |
| Farrelly et al.(14) | 2017 | USA | EA | Reviewing by staff | NA | All clinicians had to agree on removal | -39% |
| Herlihy et al.(49) | 2023 | Ireland | EA | Observations of use during procedures | 45 | <40% IUR | -35% |
| Koyle et al.(50) | 2018 | Canada | EA, LP | Observations of use during procedures; analyzing and presenting data; consensus groups | Pre-op: 45 Post-op: 60 | <50% IUR | -78% |
| Shaw et al.(51) | 2022 | USA | EA | Observations of use during procedures; consensus groups | 20 | NA | -56% |
| *Plastic surgery (n=4)* | | | | | | | |
| Dorante et al.(52) | 2023 | USA | EA | Observations of use during procedures | 37 | <20% IUR | -76% |
| Kirn et al.(53) | 2018 | USA | EA | Reviewing by staff | 240 | NA | -85% |
| Kodumuri et al.(54) | 2023 | UK | LP | NA | Pre-op: 7 Post-op: 103 | NA | -72% |
| Wood et al.(55) | 2021 | USA | EA, LP | Observations of use during procedures; analyzing and presenting data; consensus groups | 183 | NA | -40% |
| *Urology (n=1)* | | | | | | | |
| Nast et al.(15) | 2019 | USA | EA, LP | Observations of use during procedures; analyzing and presenting data; consensus groups | 24 | <20% IUR | -39% |
| *Other (n=2)* | | | | | | | |
| Toor et al.(63) | 2022 | Canada | EA | Observations of use during procedures; analyzing and presenting data; reviewing by staff | NA | NA | -35% |
| Toor et al.(48) | 2022 | Canada | EA, MP | Observations of use during procedures; reviewing by staff; linear programming | NA | NA | -69% |

***Abbreviations:*** NA = not available; NL = Netherlands; UK = United Kingdom; USA = United states of America; Post-op = postoptimization; Pre-op = pre-optimization; IUR = instrument usage rate; EA = expert analysis; LP = Lean practices; MP = Mathematial programming

**Supplementary Table S2: Effects of tray optimization on number of instruments, instrument usage and weight**

| ***Author*** | ***Year*** |  | ***Number of tray(s)*** | ***Number of instruments*** | | ***Mean instrument usage (%)*** | ***Weight per tray (kg)*** | | |
| --- | --- | --- | --- | --- | --- | --- | --- | --- | --- |
| *Breast surgery (n=3)* | | | | | | | | | |
| Holland et al.(56) | 2022 | Pre-op | 1 | 82 | -21% | NA | 8.1 | | -15% |
|  |  | Post-op | 1 | 65 |  | NA | 6.9 | |  |
| Malone et al.(57) | 2019 | Pre-op | 1 | 98 | -48% | NA | 12.3 | | -41% |
|  |  | Post-op | 1 | 51 |  | NA | 7.3 | |  |
| Schwartz et al.(58) | 2021 | Pre-op | 1 | 132 | -49% | 55% | 13.6 | | -33% |
|  |  | Post-op | 1 | 67 |  | NA | 9.1 | |  |
| *Cardiothoracic and vascular surgery (n=6)* | | | | | | | | | |
| Barua et al.(42) | 2017 | Pre-op | 1 | 119 | -34% | NA | 6.4 | -18% | |
|  |  | Post-op | 1 | 78 |  | NA | 5.2 |  |  |
| Friend et al.(40) | 2018 | Pre-op | 1 | 119 | -41% | NA | 25 | -77% | |
|  |  | Post-op | 1 | 70 |  | NA | 5.7 |  |  |
| Knowles et al.(43) | 2021 | Pre-op | 2 | 283 | -55% | 13-23% | NA | NA | |
|  |  | Post-op | 2 | 128 |  | NA | NA |  |  |
| Sanchez et al.(41) | 2023 | Pre-op | 4 | 147 | -62% | NA | NA | NA | |
|  |  | Post-op | 4 | 56 |  | NA | NA |  |  |
| Taylor et al.(44) | 2020 | Pre-op | 3 | 194 | -92% | NA | NA | NA | |
|  |  | Post-op | 1 | 16 |  | NA | NA |  |  |
| Warner et al.(45) | 2015 | Pre-op | 2 | 184 | -32% | NA | NA | NA | |
|  |  | Post-op | 2 | 126 |  | NA | NA |  |  |
| *Gynecologic surgery (n=3)* | | | | | | | | | |
| Bachmann et al.(46) | 1998 | Pre-op | 1 | 39 | -44% | NA | NA | NA | |
|  |  | Post-op | 1 | 22 |  | NA | NA |  |  |
| Harvey et al.(47) | 2017 | Pre-op | 5 | 437 | -32% | NA | NA | NA | |
|  |  | Post-op | 5 | 297 |  | NA | NA |  |  |
| Schmidt et al.(13) | 2023 | Pre-op | 3 | 154 | -29% | 55% | NA | NA | |
|  |  | Post-op | 1 | 109 |  | NA | NA |  |  |
| *Neurosurgery (n=3)* | | | | | | | | | |
| Belhouari et al.(59) | 2023 | Pre-op | 2 | 144 | -17% | NA | NA | NA | |
|  |  | Post-op | 2 | 119 |  | NA | NA |  |  |
| Farrokhi et al.(60) | 2015 | Pre-op | 3 | 421 | -75% | NA | NA | NA | |
|  |  | Post-op | 3 | 106 |  | NA | NA |  |  |
| Lunardini et al.(37) | 2014 | Pre-op | 2 | 152 | -41% | 58% | 19.1 | -41% | |
|  |  | Post-op | 1 | 89 |  | NA | 11.2 |  |  |
| *Ophthalmology (n=2)* | | | | | | | | | |
| Grodsky et al. (61) | 2020 | Pre-op | 1 | 64 | -89% | NA | 3 | -93% | |
|  |  | Post-op | 1 | 7 |  | NA | 0.22 |  |  |
| Schneider et al.(62) | 2020 | Pre-op | 17 | 374 | -21% | NA | NA | NA | |
|  |  | Post-op | 17 | 294 |  | NA | NA |  |  |
| *Orthopedics (n=8)* | | | | | | | | | |
| Adamczyk et al.(31) | 2022 | Pre-op | 8 | 163 | -49% | NA | NA | NA | |
|  |  | Post-op | 2 | 83 |  | NA | NA |  |  |
| Capra et al.(32) | 2019 | Pre-op | 30 | 531 | -57% | NA | 70.8 | -47% | |
|  |  | Post-op | 9 | 228 |  | NA | 37.3 |  |  |
| Cichos et al.(33) | 2019 | Pre-op | 11 | 792 | -55% | 23% | NA | NA | |
|  |  | Post-op | 11 | 359 |  | 52% | NA |  |  |
| Helmkamp et al.(34) | 2022 | Pre-op | 1 | 120 | -50% | NA | NA | NA | |
|  |  | Post-op | 1 | 59 |  | NA | NA |  |  |
| Hermena et al.(35) | 2021 | Pre-op | 66 | NA | -62% | NA | NA | NA | |
|  |  | Post-op | 25 | NA |  | NA | NA |  |  |
| Lonner et al.(36) | 2021 | Pre-op | 2 | 199 | -37% | 36-46% | NA | NA | |
|  |  | Post-op | 2 | 125 |  | NA | NA |  |  |
| Parker et al.(38) | 2024 | Pre-op | 1 | 106 | -64% | NA | NA | NA | |
|  |  | Post-op | 1 | 38 |  | NA | NA |  |  |
| Toor et al. (39) | 2021 | Pre-op | 1 | 88 | -42% | NA | NA | NA | |
|  |  | Post-op | 1 | 51 |  | NA | NA |  |  |
| *Otorhinolaryngology (n=10)* | | | | | | | | | |
| Chin et al.(17) | 2014 | Pre-op | 5 | 403 | -58% | 20-37% | NA | NA | |
|  |  | Post-op | 5 | 169 |  | NA | NA |  |  |
| Crosby et al. (16) | 2020 | Pre-op | 4 | 364 | -52% | NA | NA | NA | |
|  |  | Post-op | 4 | 175 |  | NA | NA |  |  |
| Dyas et al. (23) | 2018 | Pre-op | 2 | 98 | -63% | NA | 12.5 | -63% | |
|  |  | Post-op | 1 | 36 |  | NA | 4.5 |  |  |
| Fu et al.(24) | 2021 | Pre-op | 5 | 207 | -29% | 35% | NA | NA | |
|  |  | Post-op | 5 | 146 |  | NA | NA |  |  |
| Gidumal et al.(25) | 2021 | Pre-op | 3 | 189 | -64% | 17% | NA | NA | |
|  |  | Post-op | 1 | 68 |  | 47% | NA |  |  |
| John-Baptiste et al.(26) | 2016 | Pre-op | 5 | 403 | -56% | NA | NA | NA | |
|  |  | Post-op | 5 | 177 |  | NA | NA |  |  |
| Van Osch et al.(30) | 2024 | Pre-op | 1 | 18 | -44% | NA | NA | NA | |
|  |  | Post-op | 1 | 8 |  | NA | NA |  |  |
| Wannemuehler et al.(27) | 2015 | Pre-op | 1 | 52 | -54% | NA | NA | NA | |
|  |  | Post-op | 1 | 24 |  | NA | NA |  |  |
| Yalamanchi et al.(28) | 2022 | Pre-op | 4 | 313 | -28% | NA | NA | NA | |
|  |  | Post-op | 4 | 225 |  | NA | NA |  |  |
| Yoon et al.(29) | 2019 | Pre-op | 609 | 18,952 | -10% | NA | NA | NA | |
|  |  | Post-op | 609 | 17,084 |  | NA | NA |  |  |
| *Pediatric surgery (n=4)* | | | | | | | | | |
| Farrelly et al.(14) | 2017 | Pre-op | 21 | 3012 | -39% | NA | 40.5 | -32% | |
|  |  | Post-op | 9 | 1822 |  | NA | 27.7 |  |  |
| Herlihy et al.(49) | 2023 | Pre-op | NA | 49 | -35% | 56% | 3.5 | -22% | |
|  |  | Post-op | NA | 32 |  | 80% | 2.7 |  |  |
| Koyle et al.(50) | 2018 | Pre-op | 2 | 147 | -78% | NA | 6.1 | -42% | |
|  |  | Post-op | 2 | 32 |  | NA | 3.5 |  |  |
| Shaw et al.(51) | 2022 | Pre-op | 1 | 145 | -56% | NA | 8.7 | -31% | |
|  |  | Post-op | 1 | 65 |  | NA | 6.0 |  |  |
| *Plastic surgery (n=4)* | | | | | | | | | |
| Dorante et al.(52) | 2023 | Pre-op | 1 | 133 | -76% | NA | NA | | NA |
|  |  | Post-op | 1 | 32 |  | NA | NA | |  |
| Kirn et al.(53) | 2018 | Pre-op | 1 | 105 | -85% | 7% | NA | | NA |
|  |  | Post-op | 1 | 16 |  | NA | NA | |  |
| Kodumuri et al.(54) | 2023 | Pre-op | 1 | 25 | -72% | NA | NA | | NA |
|  |  | Post-op | 1 | 7 |  | NA | NA | |  |
| Wood et al.(55) | 2021 | Pre-op | 2 | 180 | -40% | 16-24% | NA | | NA |
|  |  | Post-op | 2 | 108 |  | NA | NA | |  |
| *Urology (n=1)* | | | | | | | | | |
| Nast et al.(15) | 2019 | Pre-op | 1 | 57 | -39% | 21-42% | NA | | NA |
|  |  | Post-op | 1 | 35 |  | 48-73% | NA | |  |
| *Other (n=2)* | | | | | | | | | |
| Toor et al.(63) | 2022 | Pre-op | 128 | 7,787 | -35% | NA | NA | | NA |
|  |  | Post-op | 99 | 5,044 |  | NA | NA | |  |
| Toor et al.(48) | 2022 | Pre-op | 3 | 49 | -69% | NA | NA | | NA |
|  |  | Post-op | 1 | 15 |  | NA | NA | |  |

***Abbreviations:*** NA = not available; Post-op = postoptimization; Pre-op = pre-optimization.
